# Climatic Variability and Milk Quality as Sustainability Indicators in Dairy Farming Systems of Pastaza Province, Ecuador

**DOI:** 10.3390/ani16101458

**Published:** 2026-05-09

**Authors:** Darwin Yanez Avalos, José de la Torres Moreira, Johana Delgado Lozada, Kimberley Villamarin Alvarez, Milton Montalvo Lozada, Carlos Chasipanta Chuquimarca, John Castillo Torres, Iván González-Puetate, Ronnie Mayorga Burbano, Luis Condo Plaza, Manuel Paredes Orozco, Pablo Marini, Franklin Sánchez Pila, Kleber Gallegos Guerra

**Affiliations:** 1 Fauna, Conservation and Global Health Research Group, Amazonian Regional University (Ikiam), Km 7 Via Muyuna, Muyuna Parish, Tena 150102, Ecuador; darwin.yanez@ikiam.edu.ec (D.Y.A.); johana.delgado@ikiam.edu.ec (J.D.L.); kimberley.villamarin@ikiam.edu.ec (K.V.A.); milton.montalvo@ikiam.edu.ec (M.M.L.); carlos.chasipanta@ikiam.edu.ec (C.C.C.); john.castillo@ikiam.edu.ec (J.C.T.); 2 Faculty of Veterinary Medicine, University of Guayaquil, Guayaquil 090510, Ecuador; ivan.gonzalezp@ug.edu.ec; 3 School of Agricultural and Environmental Sciences, Ibarra Campus, Pontifical Catholic University of Ecuador, Ibarra 100112, Ecuador; ramayorga@puce.edu.ec; 4 Animal Science Programme—Ecuador, Polytechnic School of Chimborazo (ESPOCH), Riobamba 060155, Ecuador; luis.condop@espoch.edu.ec (L.C.P.); patricio.paredes@espoch.edu.ec (M.P.O.); 5 Faculty of Veterinary Sciences, National University of Rosario, Rosario S2000CGK, Argentina; pmarini@unr.edu.ar; 6 Agroecology Program, Amazon Regional University (IKIAM), Tena 150102, Ecuador; franklin.sanchez@ikiam.edu.ec; 7 Faculty of Life Sciences, Graduate School, Amazon Regional University (Ikiam), Km 7 Via Muyuna, Muyuna Parish, Tena 150102, Ecuador; kleber.gallego@ikiam.edu.ec

**Keywords:** dairy systems, climatic variability, milk quality, somatic cell count, tropical livestock systems, Ecuadorian Amazon, sustainable livestock production

## Abstract

Milk production in humid tropical regions depends heavily on environmental conditions, yet little is known about how climatic variability affects milk quality in small-scale dairy systems in the Ecuadorian Amazon. This study examined the link between climatic variability and the physicochemical and microbiological quality of raw milk on dairy farms in Pastaza Province, Ecuador. Researchers collected and analyzed 127 milk samples in 2024 for fat, protein, total solids, and solids-not-fat using an automated milk analyzer. They also measured somatic cell count and total bacterial count as microbiological indicators. Climatic data, including precipitation, mean temperature, evaporation, relative humidity, cloud cover, and wind speed, were obtained from official meteorological records and analyzed using generalized linear models and multivariate analysis. The physicochemical makeup of milk remained stable despite climate change, indicating that tropical pasture-based dairy systems exhibit some productive resilience. By contrast, microbiological indicators, especially somatic cell count, varied more and were sensitive to environmental factors such as wind speed. These results show that milk composition remains stable under humid tropical conditions, whereas sanitary indicators respond more to climate variability. Better management and hygiene are crucial to maintaining sustainable dairy production systems in these environments.

## Simple Summary

Milk production in humid tropical regions is strongly influenced by environmental conditions such as rainfall, humidity, and temperature. In the Ecuadorian Amazon, dairy farming is mainly carried out by small-scale producers who depend heavily on local climatic conditions for animal health and milk production. However, limited information exists on how climatic variability affects milk quality in these production systems. This study evaluated the relationship between climatic conditions and milk quality on dairy farms in Pastaza Province, Ecuador, in the Ecuadorian Amazon. The physicochemical composition and microbiological indicators of milk were analyzed and compared with local climatic variables obtained from official meteorological archives. The results showed that the nutritional composition of milk remained relatively stable despite climatic variability, suggesting a degree of productive resilience in these pasture-based systems. In contrast, microbiological indicators related to udder health and milk hygiene showed greater variability and appeared to be more sensitive to environmental conditions. These findings highlight the need to improve sanitary management to strengthen the sustainability of dairy production in the humid tropics.

## 1. Introduction

Dairy production is fundamental for food security, the rural economy, and the sustainable development of local communities in tropical and subtropical regions. Small-scale dairy systems play a key role in providing animal-source foods. However, they often face organizational constraints in management and animal health. These systems are also more dependent on local environmental conditions [1]. In humid tropical regions, such as the Ecuadorian Amazon and especially the province of Pastaza, climate variability is pronounced. High precipitation, high relative humidity, and changes in variables such as evaporation and ambient temperature characterize the area (preprint) [2]. These conditions directly influence animal comfort, fodder availability and quality, and the hygienic and sanitary management of dairy herds [3,4]. Prolonged exposure to warm, humid environments also causes physiological stress in animals. This increases the incidence of mammary diseases and compromises the microbiological quality of raw milk [5].

The physicochemical and microbiological quality of milk is a widely used indicator for assessing production efficiency, animal health status, and management practices in dairy systems. In this context, these parameters can also be considered sustainability indicators. They reflect the ability of dairy systems to maintain product quality under climatic variability and environmental constraints. Specifically, parameters such as fat, protein, total solids, and non-fat solids (NFS) content indicate the productive stability of the system. Somatic cell count (SCC) and total bacterial count (TBC) are directly linked to mammary gland health, animal welfare, and end-product safety [6]. Several studies have shown that climatic conditions can significantly affect these milk quality indicators. For example, increased precipitation has been associated with higher SCC and TBC levels. This is due to greater environmental contamination and difficulties maintaining hygiene during milking [7]. In addition, factors such as evaporation and ambient temperature influence the water balance and heat stress in animals. These changes can alter the synthesis of milk components and reduce the productive stability of the system [8].

From a sustainability perspective, milk quality is not only a product attribute but also an integrated indicator of the strength and sustainability of local dairy systems. Notably, systems that maintain stable quality parameters despite climate variability are more adaptable, reflecting better health management and long-term production viability [9]. However, despite the relevance of these insights, few studies combine local climate variables, such as precipitation and evaporation, with physicochemical and microbiological indicators of milk in small-scale Amazonian dairy systems. This lack of data limits understanding of the true climate impact on dairy sustainability in humid tropical regions and hinders the design of evidence-based adjustment strategies [10]. Therefore, the objective of this study was to evaluate how local climate variability influences the physicochemical and microbiological quality of raw milk, as these parameters serve as sustainability indicators in Ecuadorian Amazon dairy systems.

## 2. Materials and Methods

### 2.1. Research Location

Ecuador is characterized by high climatic diversity due to its geographical location on the equator and the influence of the Andes mountain range, which generates distinct ecological regions including the Coast, Highlands, and Amazon. The Amazon region, where Pastaza Province is located, is characterized by a humid tropical climate with high annual rainfall, high relative humidity, and relatively stable temperatures throughout the year. These environmental conditions create a unique context for livestock production systems, particularly in terms of forage availability and microbial dynamics [11].

The research was conducted on dairy farms in the province of Pastaza, in the Ecuadorian Amazon, located at approximately 950 m above sea level. This area is characterized by a hot and humid climate with annual rainfall ranging from 3000 to 4000 mm, conditions that significantly influence cattle physiology and milk quality [12,13]. Pastaza borders Napo and Orellana to the north, Morona Santiago to the south, Peru to the east, and Tungurahua to the west. It is a valley of fluvial origin, with moderately fertile soils and agro-silvo-pastoral systems, where crops (maize, cassava, sugar cane, guava) and tropical pastures are combined, forming heterogeneous production systems [14,15].

**Figure 1:**
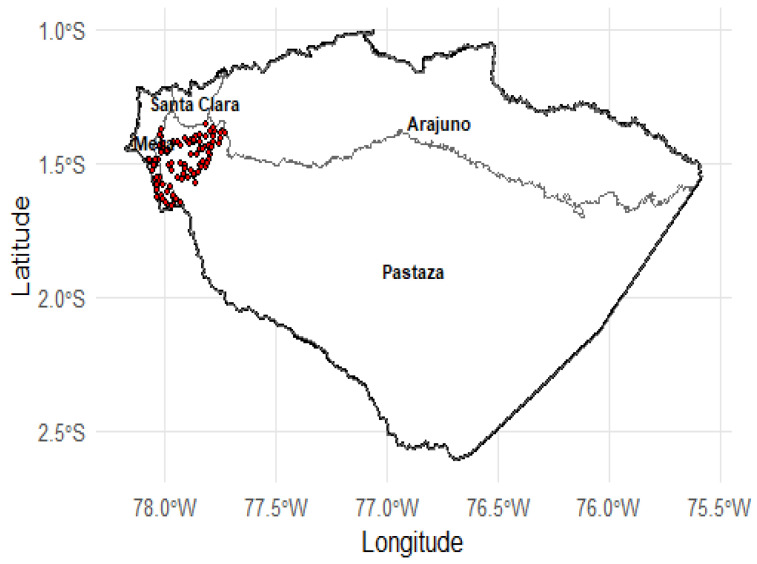
Geographical location of sampling points in the province of Pastaza, Ecuador. The map shows the district boundaries and the georeferenced sites where the samples analyzed in the study were collected.

Milk production in Ecuador is highly heterogeneous and largely dominated by small-scale production systems. According to INEN (National Institute of Statistics and Census) [16], through the Continuous Agricultural Surface and Production Survey (ESPAC), livestock production is primarily carried out in smallholder units, particularly in the Amazon region, where herd sizes are generally limited. Based on this national context, farms in the present study were classified into small (<5 animals), medium (6–10 animals), and large (>10 animals) production units. This classification does not aim to establish a universal standard but rather reflects the local production structure and management conditions observed in the study area, where even farms with 6–10 animals represent a higher level of technological and productive organization compared to smaller units. These differences contribute to gaps in technology adoption and milk hygiene quality, often associated with limitations in infrastructure, refrigeration, and access to technical resources [16,17]. The spatial distribution of the dairy farms included in the study is shown in Figure 1.

### 2.2. Sampling Protocol

The research was conducted between January and December 2024, a period characterized by high rainfall and climatic variability in the Ecuadorian Amazon [12]. A total of 127 milk samples were obtained from different dairy farms. The farms were selected using simple random sampling, establishing as inclusion criteria farms with more than five milking cows, following representativeness criteria applied in milk quality studies in tropical systems [13,17]. The total number of farms included in the study was 72, with the number of samples per farm varying according to production volume. Sampling was distributed throughout the study period, with approximately 13 samples collected per month. The cattle population consisted mainly of Brown Swiss, Holstein, and Gyr crossbreeds adapted to tropical conditions. Herd size varied among farms, reflecting the heterogeneity of smallholder production systems in the study area. Information regarding parity, stage of lactation, and individual animal characteristics was not controlled during sampling and is recognized as a limitation of the study. Therefore, independence between observations should be interpreted with caution, considering the hierarchical structure of the data (samples nested within farms and sampling periods).

Raw milk samples were collected under strict hygienic conditions, using sterile 500 mL glass bottles, and extracted directly from barrels selected at random according to the production volume of each herd, with the aim of ensuring representativeness and avoiding bias. Milking on the evaluated farms was performed manually under routine field conditions. Before milking, basic hygienic practices were applied, including udder cleaning and the use of clean utensils and containers. In addition, the equipment and materials used during milking were cleaned by the farmers according to the usual management practices of each farm. After milking, the milk was stored in bulk containers prior to sampling. All equipment used for sampling was previously sterilized and handled with gloves, a mask, and a clean gown to prevent any type of contamination that could alter the physicochemical or microbiological properties of the milk. The samples were transported to the laboratory in polystyrene boxes with refrigerants, maintaining a controlled temperature between 2 °C and 8 °C, without freezing, and were properly labelled for traceability. This procedure is based on the recommendations of the Codex Alimentarius [18], the FAO [12], and studies such as those by Mogotu [19] and Silva [11], which highlight the importance of hygiene, temperature control, and rigorous documentation in the handling of milk samples intended for laboratory analysis. Because this study was conducted under field conditions in small-scale dairy farms, milking hygiene and post-milking handling practices may have varied slightly between farms and should be considered when interpreting the microbiological results.

### 2.3. Physicochemical Analysis of Raw Cow’s Milk Samples

The physicochemical components of bovine milk, including protein, fat, total solids (TS), NFS, lactose, water percentage, freezing point, salts and density, were determined using the Lactoscan SP automatic analyzer (Apple Industries Services, La Roche Sur Foron, France—2012 by Milkotronic Ltd., Nova Zagora, Bulgaria), renowned for its accuracy in rapid raw milk analysis. To do this, 25 mL of each sample was placed in the sample holder, the equipment was set to standby mode, and the measurement was started, with the results obtained within 45 s via the digital display (IED). This procedure was performed in triplicate to ensure data reliability, following standards established in milk quality studies and instrumental protocols validated by the FAO [13], Boudalia [17] and Rodríguez & Cárdenas [14], who highlight the importance of automated methods to ensure accuracy in the nutritional characterization of milk.

### 2.4. Climate Data

The climate data used in this study were obtained from the official database of the National Institute of Meteorology and Hydrology (INAMHI) [20], the agency responsible for meteorological and climate monitoring at the national level. Records from the INAMHI weather station M0008, located in Veracruz (a local administrative parish) in Pastaza Province, were used, as they provide representative information on the environmental conditions of the dairy systems evaluated.

Climate data were collected for the period between January and December 2024, coinciding with the milk sampling period. The variables considered included precipitation (mm), mean air temperature (°C), evaporation (mm), relative humidity (%), cloud cover (octas) and wind speed (m s^−1^). These variables were selected because of their potential influence on the thermal comfort of livestock, the microbial dynamics of the environment, and the availability of forage resources in tropical dairy systems.

The daily meteorological records provided by INAMHI were processed and aggregated monthly to harmonize the climate information with the timing of milk sampling. In this process, precipitation was expressed as monthly accumulation, while the other variables were calculated as monthly averages. This procedure enabled the evaluation of the relationship between local climate variability and the physicochemical and microbiological parameters of milk analyzed in the study. The use of data from an official meteorological source ensures the reliability, standardization, and comparability of climate information, enabling integration with previous research on agricultural systems in the Amazon region.

Climatic data were aggregated at the monthly level, resulting in 12 temporal observations for the study period. Each milk sample was assigned to the corresponding monthly climatic values based on its sampling date. Therefore, climatic predictors represent shared environmental conditions within each month rather than independent observations for each individual sample.

### 2.5. Statistical Analysis

All statistical analyses were performed using the R statistical environment (version 4.3.1; R Foundation for Statistical Computing, Vienna, Austria) [21] and the integrated development environment RStudio (Posit Software, PBC) [22]. Data analysis and graphical visualization were carried out using functions from the base stats package, as well as the additional packages car (version 3.1-2) [23] and ggplot2 (version 3.4.4) [24]. Initially, a descriptive analysis of the climate and milk quality variables was performed, calculating the mean, standard deviation (SD), minimum and maximum values, and coefficient of variation (CV) to characterize the dispersion and overall behavior of the data. The normality of the continuous variables was assessed using the Shapiro–Wilk test. Given that this test can be highly sensitive in moderate to large sample sizes (*n* > 100), the homogeneity of variances between months was examined using Levene’s test, using the mean as a reference point to reduce the influence of non-normal distributions. The SCC and TBC variables showed positive asymmetry, so a base-10 logarithmic transformation [log10(x + 1)] was applied to improve normality and stabilize the variance before inferential analyses. Although the somatic cell score transformation proposed by Ali and Shook [25] is widely used in dairy science, it is primarily intended for genetic and longitudinal analyses. In the present study, the log10 transformation was considered more appropriate due to its simplicity, robustness, and widespread use in studies evaluating environmental and microbiological data under field conditions.

To evaluate the association between climatic variables and milk quality parameters, general linear models (GLM) with Gaussian distribution and identity link function were fitted. The climate variables included as predictors were precipitation (mm), evaporation (mm), mean temperature (°C), relative humidity (%), cloud cover (octas) and wind speed (m/s). The parish was incorporated as a categorical fixed factor to control spatial heterogeneity.

The general model took the following form:

Y_i = β_0 + β_1 Prec_i + β_2 Evap_i + β_3 Temp_i + β_4 RH_i + β_5 Nub_i + β_6 Wind_i + β_7 Parish_i + ε_i

where Y_i represents the response variable (log_SCC, log_TBC, or physicochemical parameter), β_0 is the intercept, β_1–β_7 are the regression coefficients, and ε_i is the random error term. Prec corresponds to precipitation (mm), Evap to evaporation (mm), Temp to mean air temperature (°C), RH to relative humidity (%), Nub to cloud cover (octas), Wind to wind speed (m s^−1^), and Parish represents the categorical spatial factor included in the model.

The model assumptions were verified using graphical residual analysis, including assessment of homoscedasticity and normality of residuals. Multicollinearity among predictors was examined using the Variance Inflation Factor (VIF), with values greater than 5 considered indicative of potential collinearity. Although moderate levels of collinearity were detected among some climatic variables, all predictors were retained in the final models due to their ecological and biological relevance. In humid tropical systems, climatic variables such as precipitation, humidity, evaporation, and temperature are inherently interrelated and represent a complex environmental context. Therefore, the models were interpreted from an explanatory rather than predictive perspective.

The goodness of fit of the model was evaluated using the coefficient of determination (R^2^), adjusted R^2^, global F statistic, and Akaike information criterion (AIC). A statistical significance level of *p* < 0.05 was established. Given that climatic data were aggregated at the monthly level, statistical inferences were interpreted with caution, recognizing that climatic variability was effectively represented by a limited number of temporal units. Therefore, the independence of observations is conditioned by the temporal structure of the data, which may influence the estimation of model parameters and statistical significance. Additionally, the predicted marginal effects for the selected climate variables were estimated, keeping the other predictors constant at their mean values, to visualize the magnitude and direction of the modelled associations.

## 3. Results

### 3.1. Climatic Characterization of the Study Period

**Figure 2:**
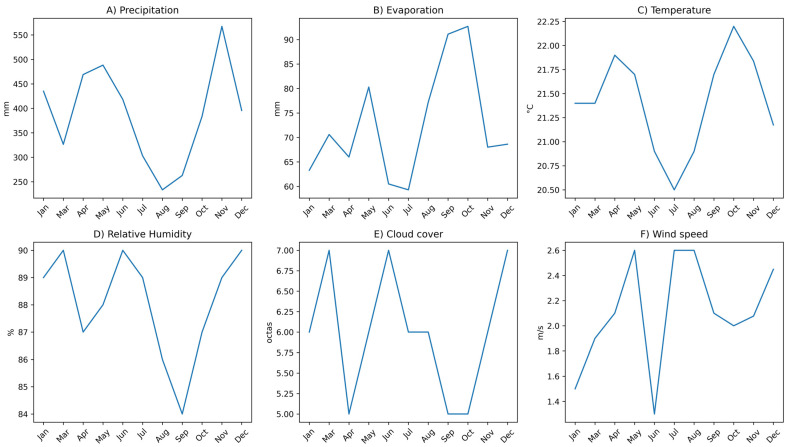
Monthly variation of climatic variables during the study period in Pastaza Province, Ecuador. Panels show (**A**) precipitation (mm), (**B**) evaporation (mm), (**C**) mean temperature (°C), (**D**) relative humidity (%), (**E**) cloud cover (octas), and (**F**) wind speed (m s^−1^). Climatic data were obtained from the National Institute of Meteorology and Hydrology (INAMHI).

Descriptive statistics, including measures of central tendency (mean), dispersion (standard deviation), and relative variability (coefficient of variation, CV), for climatic variables are presented in Table 1, while their monthly temporal behavior is illustrated in Figure 2. During the evaluated period (n = 12 observations), precipitation showed a high mean value and the highest coefficient of variation among the variables analyzed, indicating marked dispersion. The precipitation range exhibited considerable amplitude, reflecting notable differences between months with the lowest and highest rainfall accumulation. Evaporation showed intermediate variability, with monthly fluctuations visible in Figure 1, although with a lower relative amplitude than that of precipitation. In contrast, temperature exhibited the lowest relative dispersion, remaining within a narrow range throughout the study period; this thermal stability is reflected in the nearly horizontal monthly trend shown in Figure 1.

Relative humidity remained consistently high, with moderate variations between months, while cloud cover showed limited fluctuations throughout the period evaluated. Figure 2 shows that the monthly variations in these variables followed relatively stable patterns, unlike precipitation. Taken together, the data presented in Table 1 and Figure 2 show a climate regime characterized by high precipitation, high ambient humidity, and thermal stability, with more pronounced interannual fluctuations in the hydrological components than in the thermal components.

### 3.2. Physicochemical and Microbiological Milk Quality

The physicochemical parameters showed moderate variability, with coefficients of variation lower than those observed in the microbiological variables (Table 2). Fat and protein showed relative stability, while TS and SNF showed variations associated with environmental conditions. Microbiological indicators SCC and TBC showed high dispersion, with positive asymmetric distribution, which justified logarithmic transformation for inferential analysis.

### 3.3. Climatic Effects on Milk Microbiological Indicators

The results of the general linear model for log_SCC are presented in Table 3; the analysis revealed significant effects associated with both spatial factors and a specific climatic variable. Wind speed showed a significant positive association with log_SCC (*p* < 0.05), while evaporation showed a marginal trend towards statistical significance. Significant differences were also observed between parishes, indicating spatial heterogeneity in somatic cell levels.

The adjusted model for log_SCC showed limited associations with climatic variables. Wind speed was the only variable that exhibited a statistically significant effect (*p* < 0.05). Evaporation showed a marginal trend towards statistical significance, whereas precipitation and mean temperature did not.

**Figure 3:**
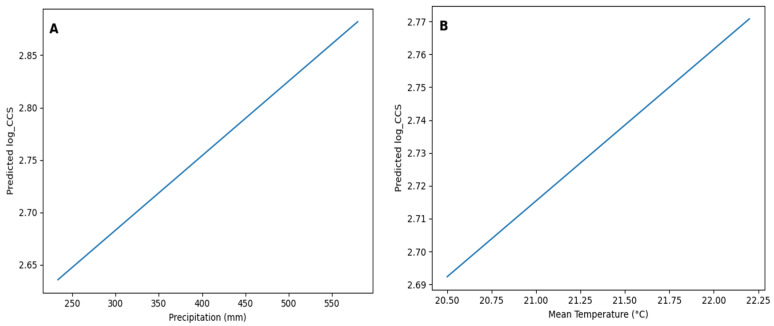
Predicted marginal effects of (**A**) precipitation (mm) and (**B**) mean temperature (°C) on log-transformed somatic cell count (log_SCC), estimated from the fitted general linear model. Other predictors were held at their mean values.

VIF values indicated moderate to high multicollinearity among some climatic predictors. Specifically, relative humidity showed the highest VIF value (>10), while cloud cover, evaporation, and temperature also exhibited elevated values (>5). These results suggest that some climatic variables are interrelated, as expected under tropical conditions. Despite this, all variables were retained in the model due to their biological relevance and to preserve the integrative nature of the climatic system under study. Finally, the predicted marginal effects of selected climatic variables on log-transformed somatic cell count (log_SCC) are illustrated in Figure 3.

In contrast, the adjusted model for log_TBC, shown in Table 4, did not show statistically significant associations with the evaluated climatic variables (*p* > 0.05). Although numerical variations were recorded in the coefficients associated with precipitation and average temperature, these did not reach significance in the multivariate model. The differences between parishes also did not show consistent effects on this microbiological parameter. The model fit statistics, including the coefficient of determination (R^2^) and adjusted R^2^, indicated moderate explanatory power for log_SCC and limited explanatory power for log_TBC (Table 3 and Table 4).

### 3.4. Climatic Influence on Physicochemical Composition

The results of the general linear models adjusted for physicochemical variables are presented in Table 5. In general, the climatic variables evaluated showed only limited associations with milk compositional components. For fat and protein, the estimated coefficients for precipitation, evaporation, mean temperature, relative humidity, cloud cover, and wind speed did not show statistically significant effects in the multivariate model (*p* > 0.05). Similarly, the models adjusted for TS and NFS showed coefficients of low magnitude and without consistent statistical significance.

For pH, titratable acidity, density corrected to 15 °C, and cryoscopy, the models showed moderate explanatory power, with low R^2^ values and no significant climatic associations for most predictors. However, in some models, differences associated with the parish factor were observed, suggesting spatial heterogeneity in certain compositional parameters, independent of climatic variables. Overall, the results presented in Table 5 indicate that the variability of the physicochemical parameters was poorly explained by the climatic predictors considered.

The goodness-of-fit statistics for the fitted models are presented in Table 6. The model for log_SCC showed moderate explanatory power and was statistically significant, whereas the model for log_TBC showed limited explanatory power and was not statistically significant.

**Figure 4:**
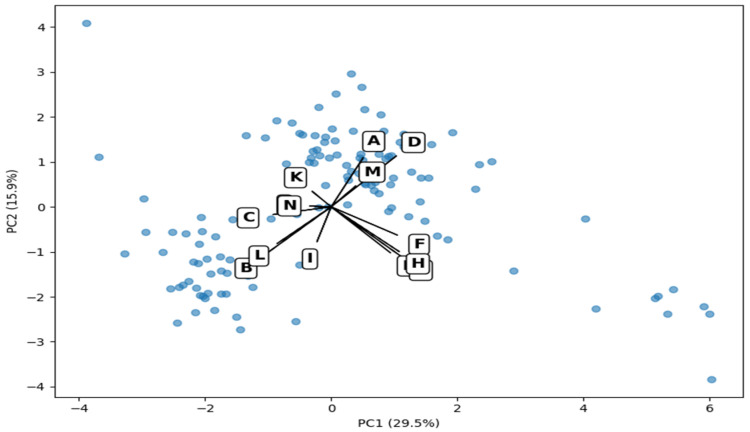
Principal Component Analysis (PCA) biplot integrating climatic and milk quality variables.

A Principal Component Analysis (PCA) was conducted to evaluate the integrated structure of climatic and milk quality variables. The first two principal components accounted for 45.4% of the total variance (PC1 = 29.5%; PC2 = 15.9%). This moderate explained variance suggests a high level of complexity in the dataset. This complexity likely stems from the inclusion of heterogeneous variables (physicochemical, microbiological, and climatic). Therefore, PCA was primarily used as an exploratory tool to visualize potential patterns and relationships among variables, rather than for strong dimensional reduction. Loadings indicated different contributions of compositional and microbiological parameters along PC1. Climatic variables were strongly associated with PC2. Figure 4 illustrates the spatial distribution of samples and variable loadings.

Principal Component Analysis (PCA) biplot showing the integration of climatic and milk quality variables. Variables were standardized prior to analysis. Arrows represent loadings of each variable on the first two principal components (PC1 and PC2), which explain 29.5% and 15.9% of the total variance, respectively. Letters correspond to variables as follows: (A) Precipitation (mm); (B) Evaporation (mm); (C) Mean Temperature (°C); (D) Relative Humidity (%); (E) Fat (%); (F) Protein (%); (G) Total Solids (%); (H) Solids-Non-Fat (%); (I) pH; (J) Titratable Acidity; (K) Density (15 °C); (L) Cryoscopy; (M) Somatic Cell Count; and (N) Total Bacterial Count.

## 4. Discussion

### 4.1. Climate Dynamics in Amazonian Dairy Systems

The marked rainfall variability observed during the study period is consistent with the hydroclimatic dynamics described for humid Amazonian ecosystems, in which precipitation is the primary environmental driver of agricultural systems. In the Ecuadorian Amazon, rainfall patterns tend to show inter-monthly fluctuations associated with regional atmospheric circulation and the interaction between moist air masses from the Atlantic and the Andean-Amazonian topography [26]. This behavior contrasts with that of temperate regions, where thermal variability has a greater influence on production systems than water variability.

The high precipitation dispersion observed in this study suggests an environmental dynamic in which periods of higher water accumulation could indirectly modify soil conditions, drainage, and the microenvironment in areas used for milking and animal rest. In tropical dairy systems, rainfall variability has been documented to influence forage quality, soil compaction, and environmental microbial load, thereby impacting health and production parameters [27]. However, unlike seasonal systems, in the humid tropics, the absence of a prolonged dry season favors the persistence of vegetation cover throughout the year, mitigating potential extreme nutritional effects. The relative thermal stability recorded during the period evaluated is consistent with the typical behavior of equatorial zones, where the annual temperature range is usually lower than that observed in mid-latitudes (preprint) [28]. This stability factor reduces the likelihood of pronounced seasonal heat stress, a phenomenon widely documented as a critical factor in the decline of milk production and quality in subtropical and temperate regions [29,30]. In contrast, in Amazonian ecosystems, the thermal component tends to play a secondary role compared to factors related to humidity and precipitation.

Persistently high levels of relative humidity, combined with relatively stable cloud cover, reflect atmospheric conditions that favor environments with high microbial loads. Several studies have shown that in humid tropical systems, environmental humidity is an indirect determinant of the dynamics of environmental pathogens associated with mastitis [31,32]. From a systemic perspective, the climate regime described suggests that Amazonian dairy systems operate under relatively constant temperature conditions, but with significant water variability [33,34]. This combination can create a scenario in which productive resilience depends more on the ability to manage fluctuations in humidity and drainage than on adaptations to temperature extremes.

Additionally, under projected climate change scenarios for the Amazon basin, regional models anticipate changes in precipitation patterns rather than drastic increases in average annual temperature [35]; in this sense, understanding rainfall dynamics and their interaction with local dairy systems is essential to anticipate impacts on productive sustainability. Taken together, the climate characterization not only describes the environmental context of the study but also establishes the ecological framework for interpreting microbiological and physicochemical results, particularly in relation to indicators of resilience and sustainability in tropical dairy systems.

### 4.2. Physicochemical and Microbiological Quality of Milk in Humid Tropical Systems

The moderate variability observed in physicochemical parameters suggests that milk composition in Amazonian dairy systems exhibits a degree of structural stability despite the environmental fluctuations described. In tropical systems based on permanent grazing, the relatively constant availability of forage biomass throughout the year tends to reduce the compositional fluctuations that characterize seasonal systems in temperate zones [36]. This relative stability in fat and protein is consistent with research conducted in dual systems in Latin America, where milk composition showed less sensitivity to moderate climatic variations than health indicators [37].

TS and NFS, although showing slightly higher variability, continue to reflect an integrative behavior of the animal’s nutritional and metabolic status. In humid tropical environments, forage quality can vary with rainfall intensity and plant growth rate, which could explain the observed variations in these structural fractions [38]. However, the magnitude of these variations suggests that the production system maintains a buffering capacity against short-term environmental changes.

In contrast, microbiological indicators exhibited greater dispersion and positive asymmetric distribution, a pattern commonly reported in studies of SCC and TBC in tropical dairy systems [39]. This skewed distribution reflects the biological nature of inflammatory and microbial contamination processes, in which specific episodes of mastitis or hygiene failures generate extreme values that increase total variability [40]. The logarithmic transformation applied in inferential analysis is methodologically consistent with international standards for the statistical treatment of microbiological data in animal production [41]. The observed SCC and TBC values should be interpreted in accordance with established health standards for raw milk. In Ecuador, Ecuadorian Technical Standard NTE INEN 9:2012 [42] establishes quality criteria for raw milk intended for industrial processing, including microbiological and physicochemical parameters that allow its health suitability to be assessed. In small-scale tropical dairy systems, particularly in humid regions such as the Amazon, greater variability in microbiological indicators is common due to environmental factors, management practices, and limited sanitary infrastructure. Therefore, the average SCC value observed in this study (613 × 10^3^ cells mL^−1^) suggests the presence of sanitary challenges in the systems evaluated, which has been frequently reported in small-scale tropical dairy systems.

The greater dispersion in SCC with respect to compositional parameters suggests that the sanitary components of the system are more sensitive to microenvironmental and management variations than to structural changes in diet. In humid tropical regions, the combination of high environmental humidity, saturated soils and the constant presence of organic matter creates conditions conducive to the persistence of environmental pathogens, particularly those associated with environmental mastitis [43], so microbiological variability can be interpreted as a dynamic indicator of the health status of the system rather than a simple statistical fluctuation.

From a productive sustainability perspective, the coexistence of compositional stability and microbiological variability suggests that the systems evaluated are nutritionally resilient but remain vulnerable to health risks. This differentiation is relevant, as the sustainability of dairy systems depends not only on milk production or composition, but also on animal health, hygienic quality, and the ability to adapt to environmental pressures [44]. Compared with studies conducted in subtropical regions, studies in temperate regions have documented that severe heat stress tends to simultaneously affect production, composition, and health parameters [45,46]. In contrast, in the humid Amazonian tropics, thermal stability could explain why physicochemical parameters remain relatively constant, while environmental humidity primarily affects microbiological dynamics.

The difference between compositional stability and microbiological dispersion highlights the need to assess multiple milk quality dimensions for sustainability in tropical systems. Physicochemical quality may show nutritional consistency. Microbiological indicators reflect sanitary and environmental conditions.

### 4.3. Climatic Effects on Microbiological Indicators of Milk

The results of the general linear model indicate that climatic variability has different effects on the microbiological indicators of milk, particularly on SCC, whereas TBC shows a less dependent response to the included climatic predictors.

The positive association observed between wind speed and log_SCC suggests indirect mechanisms related to the environmental dynamics of the production system. In tropical dairy systems, natural ventilation can modify particle dispersion, organic material accumulation, and exposure to environmental pathogens, all of which influence the incidence of subclinical mastitis [47]. The literature has documented that somatic cell counts are particularly sensitive to microenvironmental conditions affecting under health, rather than intrinsic thermal variations [48].

The marginal trend observed in evaporation could reflect complex interactions between environmental water balance and microbial persistence on contact surfaces. In humid environments, small variations in evaporation can alter the drying conditions of the floor and resting areas. This indirectly influences the environmental bacterial load [49], suggesting that the climatic component acts in conjunction with management factors [50].

Significant differences between parishes reveal spatial heterogeneity in SCC levels, suggesting that structural variables, such as milking practices, infrastructure, drainage, and sanitary management, can modulate the system’s sensitivity to environmental conditions. Studies conducted in Latin America have shown that spatial variability in SCC often exceeds climatic variability when management practices differ [51,52]. In contrast, the absence of significant associations between climatic variables and log_TBC suggests that total bacterial load depends more on post-milking processes and the hygienic management chain than on macroclimatic variability. TBC is strongly influenced by cleaning practices, water quality, cooling times, and storage conditions, factors that can dampen or amplify the impact of the external climate [53,54].

The difference in explanatory power between the log_SCC and log_TBC models is consistent with the biological nature of both indicators. While SCC reflects an animal’s physiological response to inflammatory processes and is therefore sensitive to environmental conditions that favor pathogen development, TBC is an indicator more directly linked to hygiene and handling practices [55]; this distinction is key for a systemic assessment. From a sustainability perspective, the results suggest that Amazonian dairy systems are more vulnerable in terms of under health than in terms of general hygiene control, which appears to be relatively independent of the climatic fluctuations evaluated. In practical terms, this implies that climate change adaptation strategies should prioritize strengthening environmental mastitis control measures in contexts of high humidity and water variability. Under projected climate change scenarios for the Amazon, where changes in precipitation patterns are anticipated rather than extreme increases in average temperature [56], humidity, and ventilation dynamics could become an even more relevant determinant of udder health [57]. Therefore, systematic SCC monitoring can serve as a sensitive indicator of environmental pressure and the health resilience of the production system [58]. Taken together, the results support the idea that microbiological indicators do not respond homogeneously to climate variability but rather reflect the interaction between environmental conditions and management practices, which should be considered when using microbiological quality as an indicator of sustainability in humid tropical systems.

### 4.4. Climatic Influence on Physicochemical Composition

In temperate regions, milk composition tends to show marked seasonality, associated with changes in forage availability and quality, as well as pronounced temperature variations [59]. However, in Amazonian ecosystems characterized by a low annual temperature range and continuous pasture availability, milk composition tends to remain more consistent throughout the year [60], which is consistent with the stability observed in fat and protein in the present study.

TS and NFS, although they showed moderate coefficients of determination in some models, did not show robust climatic associations. These components represent structural fractions of milk, influenced mainly by nutritional factors, the animal’s physiological status and genetics, rather than by immediate macroclimatic variations. Several recent studies indicate that milk composition is determined by interactions between genetics, stage of lactation, nutrition, and production management, while environmental factors exert indirect or secondary effects on these components [61,62]. In tropical grazing systems, the relatively constant supply of plant biomass can help stabilize nutrient intake and buffer the potential effects of water variability on milk nutritional composition, thereby reducing the sensitivity of parameters such as TS and NFS to short-term climatic fluctuations.

The physicochemical parameters related to stability and technological quality, pH, titratable acidity, corrected density, and cryoscopy, also showed low explanatory power in relation to the climatic variables included. This finding suggests that these indicators are more closely linked to internal metabolic processes and management practices than to direct external environmental conditions. The spatial heterogeneity observed in some models reinforces the hypothesis that structural differences between parishes, such as milking infrastructure or health management, may have greater explanatory weight than climatic variability.

Therefore, the limited influence of climate on physicochemical composition can be interpreted as an indicator of the system’s nutritional resilience. Resilience, understood as the capacity of productive systems to maintain structural and productive functions in the face of moderate environmental disturbances, has been widely described in the literature on sustainable agricultural systems. Small-scale tropical livestock systems based on extensive grazing often have adaptation mechanisms that buffer climatic variation through forage diversity, management flexibility, and physiological adaptation of livestock [63,64]. In this context, the compositional stability observed suggests that the systems evaluated have buffering mechanisms against the environmental fluctuations typical of the humid tropics.

Multivariate analysis using PCA enabled integrating climatic and productive dimensions into a systemic structure. The partial separation between compositional and microbiological variables in the space of the first two principal components indicates that milk quality does not respond to a single environmental gradient, but rather to the interaction of multiple factors. The stronger alignment of climatic variables and PC2 suggests that the environmental dimension explains a secondary but distinct portion of the total variability. The cumulative variance of 45.4% explained by PC1 and PC2 is consistent with the multifactorial nature of complex biological systems, in which physiological, environmental, and management variables interact simultaneously and jointly contribute to the variability observed in production systems [65,66]. In similar studies in tropical dairy systems, the principal components associated with milk composition tend to be grouped into axes distinct from those related to health, environmental, or management factors, reflecting the relative independence of milk quality’s structural variables from the microenvironmental pressures of the production system [67].

Taken together, the results suggest that the physicochemical composition of milk in Amazonian systems is relatively robust to observed climatic variability, whereas microbiological indicators are more sensitive to environmental conditions. This differentiation has important implications for sustainability assessment, as it indicates that nutritional stability can coexist with health vulnerability in contexts of high environmental humidity. In future climate change scenarios, projections for the Amazon point to changes in precipitation patterns rather than extreme temperature increases [30].

### 4.5. Limitations

A limitation of this study is the presence of moderate collinearity among climatic predictors, which may influence the stability of individual regression coefficients. However, given the ecological interdependence of climatic variables in humid tropical environments, retaining these predictors allowed for a more comprehensive representation of environmental conditions. Future studies may explore more parsimonious models to further improve interpretability.

## 5. Conclusions

The dairy systems studied in the Ecuadorian Amazon operate in a climate with high rainfall, high humidity, and little annual change in temperature. This pattern aligns with the typical conditions in humid Amazonian ecosystems and significantly shapes how dairy farms operate and the surrounding environment.

The chemical makeup of milk stayed stable despite changes in climate during the study. This suggests that tropical, pasture-based dairy farms are somewhat resilient to moderate environmental changes. This stability may be due to the steady supply of forage in humid tropical areas.

In contrast, microbiological indicators, particularly somatic cell count, showed greater sensitivity to environmental factors and spatial heterogeneity between production systems. This suggests that microenvironmental conditions and health management practices play a key role in the system’s health dynamics, and that monitoring these indicators can help assess sustainability in tropical dairy systems.

## Figures and Tables

**Table 1:** Descriptive statistics of climatic variables during the study period (n = 12).

Climatic Variable	*n*	Mean ± SD	Min–Max	CV (%)
Precipitation [mm]	12	378.11 ± 101.31	233.5–579.7	26.79
Temperature [°C]	12	21.50 ± 0.43	20.5–22.2	1.99
Evaporation [mm]	12	75.73 ± 12.94	57.6–92.7	17.08
Relative Humidity [%]	12	87.52 ± 2.38	84.0–90.0	2.72
Cloud Cover [octas]	12	5.87 ± 0.82	5.0–7.0	13.9
Wind Speed [m s^−1^]	12	2.11 ± 0.40	1.3–2.8	18.93

**Table 2:** Descriptive statistics of physicochemical and microbiological milk quality parameters (*n* = 127).

Milk Quality Variable	*n*	Mean ± SD	Min–Max	CV (%)
Fat (%)	127	3.712 ± 0.374	2.76–4.93	10.07
Protein (%)	127	3.282 ± 0.314	2.08–4.24	9.57
Total Solids (%)	127	12.601 ± 0.775	10.21–15.41	6.15
Solids Non-Fat (%)	127	8.895 ± 0.553	7.12–12.15	6.22
pH	127	6.559 ± 0.071	6.5–6.8	1.08
Acidity	127	0.172 ± 0.008	0.15–0.19	4.4
Density (°L at 15 °C)	127	28.823 ± 1.046	22.8–32.5	3.63
Cryoscopic point (°C)	127	−0.553 ± 0.024	−0.601–−0.512	4.31
SCC (×10^3^ cells mL^−1^)	127	613.559 ± 315.152	7.0–1757.0	51.36
TBC (×10^3^ CFU mL^−1^)	127	5965.677 ± 8649.060	67.0–61,109.0	144.98

SD = standard deviation; CV = coefficient of variation; Density (°L at 15 °C) = milk density expressed in lactodensimeter degrees measured at 15 °C.; SCC = somatic cell count expressed as cells mL^−1^; TBC = total bacterial count expressed as colony-forming units per milliliter (CFU mL^−1^).

**Table 3:** General Linear Model evaluating the effect of climatic variables and parish on log10-transformed somatic cell count (log10(SCC + 1)).

Term	β	SE	T	*p*-Value	95% CI
Intercept	7.134	4.96	1.44	0.153	−2.692–16.959
El Triunfo	−0.239	0.126	−1.90	0.06	−0.488–0.010
Fatima	0.238	0.158	1.51	0.135	−0.075–0.551
Puyo	−0.236	0.106	−2.22	0.029	−0.447−0.025
Shell	0.036	0.121	0.3	0.767	−0.203–0.275
Veracruz	−0.027	0.128	−0.21	0.832	−0.281–0.227
Precipitation (mm)	0.0007	0.0007	1.04	0.3	−0.0006–0.0021
Evaporation (mm)	−0.0140	0.0071	−1.97	0.051	−0.0281–0.0001
Temperature (°C)	0.046	0.18	0.26	0.798	−0.310–0.402
Relative Humidity (%)	−0.069	0.052	−1.34	0.183	−0.171–0.033
Cloud cover (octas)	0.163	0.122	1.34	0.183	−0.078–0.404
Wind Speed (m s^−1^)	0.247	0.115	2.14	0.034	0.019–0.475

β = regression coefficient; SE = standard error; CI = confidence interval. SCC values were log10-transformed prior to analysis [log10(SCC + 1)]. El Triunfo, Fatima, Puyo, Shell and Veracruz correspond to the parishes included in the model as categorical spatial factors.

**Table 4:** General Linear Model evaluating the effect of climatic variables and parish on log10-transformed total bacterial count (log10(TBC + 1)).

Term	β	SE	t	*p*-Value	95% CI
Intercept	−2.187	8.766	−0.25	0.803	−19.552–5.177
El Triunfo	0.034	0.222	0.15	0.877	−0.406–0.475
Fatima	−0.518	0.279	−1.86	0.066	−1.071–0.035
Puyo	−0.190	0.188	−1.01	0.316	−0.562–0.183
Shell	0.175	0.213	0.82	0.414	−0.248–0.598
Veracruz	0.263	0.227	1.16	0.248	−0.186–0.712
Precipitation (mm)	0.00054	0.00121	0.44	0.658	−0.00186–0.00293
Evaporation (mm)	−0.00315	0.01256	−0.25	0.802	−0.02802–0.02172
Temperature (°C)	0.321	0.317	1.01	0.314	−0.308–0.950
Relative Humidity (%)	−0.0078	0.0912	−0.09	0.932	−0.188–0.173
Cloud cover (octas)	−0.0636	0.2149	−0.30	0.768	−0.489–0.362
Wind Speed (m s^−1^)	−0.115	0.204	−0.56	0.574	−0.518–0.288

β = regression coefficient; SE = standard error; CI = confidence interval. TBC values were log10-transformed prior to analysis [log10(TBC + 1)]. El Triunfo, Fátima, Puyo, Shell and Veracruz correspond to the parishes where dairy farms were located and were included in the model as categorical spatial factors.

**Table 5:** General Linear Model results for physicochemical milk quality variables.

Dependent Variable	R^2^	Adj. R^2^	*p* (Model)
Fat (%)	0.2405	0.1678	0.0006
Protein (%)	0.3615	0.3005	<0.001
Total Solids (%)	0.5072	0.4601	<0.001
Non-Fat Solids (%)	0.5841	0.5443	<0.001
pH	0.1883	0.1106	0.0094
Acidity	0.1608	0.0805	0.0341
Density (g/mL at 15 °C)	0.1081	0.0228	0.2522
Crioscopic point (°C)	0.4481	0.3953	<0.001

R^2^ indicates the coefficient of determination, representing the proportion of variability explained by the model. Adj. R^2^ corresponds to the adjusted coefficient of determination, which corrects for the number of predictors included in the model. The *p*-value indicates the statistical significance of the overall model.

**Table 6:** Goodness-of-fit statistics for generalized linear models evaluating the effect of climatic variables on milk quality parameters.

Model	R^2^	Adj. R^2^	F-Statistic	*p*-Value	AIC
log (SCC)	0.126	0.082	2.88	0.012	106.8
log (TBC)	0.096	0.050	2.11	0.056	246.7

R^2^: coefficient of determination; Adj. R^2^: adjusted coefficient of determination; AIC: Akaike information criterion.

## Data Availability

Data is contained within the article.

## Abbreviations

The following abbreviations are used in this manuscript:

GLM	General Linear Model
PCA	Principal Component Analysis
SCC	Somatic Cell Count
TBC	Total Bacterial Count
INHAMI	Instituto Nacional de Meteorología e Hidrología
INEN	Instituto Ecuatoriano de Normalización
